# Paclitaxel- and Sirolimus-Coated Drug-Coated Balloons versus Drug-Eluting Stents in Acute Myocardial Infarction: A Comprehensive Systematic Review and Meta-Analysis

**DOI:** 10.5812/ijpr-164739

**Published:** 2026-02-08

**Authors:** Zhengmin Zhang, Gang Yu, Rong Guo

**Affiliations:** 1 Cardiovasology, Dunhuang Hospital, Dunhung, China

**Keywords:** Acute Myocardial Infarction, Percutaneous Coronary Intervention, Drug-Coated Balloon, Paclitaxel, Sirolimus, Drug-Eluting Stent, Dual Antiplatelet Therapy, Meta-Analysis

## Abstract

**Context:**

Acute myocardial infarction (AMI) requires rapid yet durable coronary reperfusion. Drug-eluting stents (DES) are the standard for percutaneous coronary intervention (PCI) but pose long-term challenges such as late stent thrombosis and extended dual antiplatelet therapy (DAPT). Drug-coated balloons (DCBs), using paclitaxel or newer sirolimus formulations, deliver antiproliferative therapy without leaving a permanent scaffold, offering a potentially safer, stent-free option.

**Objectives:**

To evaluate the efficacy and safety of DCBs versus DES in AMI, with emphasis on drug platform (paclitaxel vs. sirolimus) and specific outcomes.

**Methods:**

This systematic review and meta-analysis followed PRISMA 2020 guidelines. Databases including PubMed, Embase, Web of Science, Cochrane Library, and ClinicalTrials.gov were searched from inception through 15 January 2025, according to a prospectively archived protocol on the Open Science Framework. Thirty-four eligible studies (14 randomized controlled trials (RTCs), 20 observational cohorts) comprising 19,642 participants and 2,313 major adverse cardiovascular events (MACE) were analyzed. Dichotomous outcomes were synthesized as pooled risk ratios (RRs) with 95% confidence intervals (CIs) using a random-effects (DerSimonian-Laird) model; heterogeneity was quantified by I², τ², and H² statistics.

**Results:**

Drug-coated balloons were associated with a 15% relative reduction in MACE compared with DES (RR = 0.85, 95% CI: 0.73 - 0.99, I² = 63.2%). Rates of myocardial infarction (MI) (RR = 0.93, 95% CI: 0.78 - 1.11), target lesion revascularization (TLR) (RR = 0.88, 95% CI: 0.71 - 1.07), and cardiovascular mortality (RR = 0.97, 95% CI: 0.81 - 1.16) were comparable between groups. Late stent thrombosis was significantly lower with DCBs (RR = 0.64, 95% CI: 0.42 - 0.97). Most pooled evidence involved paclitaxel-coated balloons, while sirolimus-coated DCB data, though directionally consistent, remain limited by smaller sample size and shorter follow-up.

**Conclusions:**

This meta-analysis suggests that DCBs represent a feasible, stent-free PCI strategy in AMI, providing comparable safety and efficacy to DES with a potential advantage in reducing late thrombosis. Device selection should be guided by lesion complexity, bleeding risk, and DAPT tolerance. Further large-scale, head-to-head randomized trials, particularly involving sirolimus-coated DCBs, are warranted to validate these findings.

## 1. Introduction

Acute myocardial infarction (AMI) remains a leading cause of global morbidity and mortality, precipitated by abrupt atherothrombotic coronary occlusion and resultant ischemia that can progress to irreversible injury and impaired cardiac function if reperfusion is delayed (1). Clinically, AMI is classified as ST‐segment elevation myocardial infarction (STEMI) or non–ST‐segment elevation myocardial infarction (NSTEMI), each requiring rapid diagnosis and timely reperfusion to minimize infarct size and preserve ventricular function (2).

Primary percutaneous coronary intervention (PPCI) is the standard of care for AMI, typically combining balloon dilation with stent implantation (3, 4). Drug-eluting stents (DES) reduce neointimal hyperplasia and restenosis relative to bare-metal stents by delivering antiproliferative therapy (5). However, the permanent metallic scaffold can delay endothelial recovery and sustain late or very late stent thrombosis risk, concerns that may be accentuated in high thrombus burden settings (e.g., STEMI) and among elderly or diabetic patients, thereby mandating prolonged dual antiplatelet therapy (DAPT) with attendant bleeding trade-offs and periprocedural management challenges (6, 7).

Drug-coated balloons (DCBs) offer a stent-free alternative, delivering an antiproliferative agent during a brief (≈ 30 - 60 s) inflation without leaving a permanent implant (8). By preserving native vessel architecture, DCBs may reduce chronic vascular inflammation, obviate a nidus for late thrombosis, and enable shorter DAPT, features attractive in patients with elevated bleeding risk, small vessels, bifurcations, or scenarios where future surgical access is anticipated. Paclitaxel-coated balloons are the most extensively studied; their high lipophilicity facilitates rapid arterial uptake and sustained antiproliferative effect via microtubule stabilization and smooth-muscle growth inhibition (9). In response to healing concerns associated with paclitaxel in peripheral contexts, sirolimus-based DCBs have been developed. Sirolimus inhibits the mammalian target of rapamycin (mTOR), promoting cell-cycle arrest and anti-inflammatory effects; although less lipophilic, modern carriers (e.g., nanoparticles, phospholipid matrices) enhance tissue transfer and retention during short inflations (10). Preclinical data suggest sirolimus may favor more balanced inhibition of neointimal proliferation with supportive endothelial recovery, a distinction relevant in high-risk cohorts.

Early clinical experience with sirolimus-coated platforms (e.g., MagicTouch, Selution SLR, BioPath) has been encouraging in de-novo lesions and in patients at high bleeding risk; nonetheless, most AMI-specific evidence arises from relatively small randomized or observational cohorts with limited follow-up (11-15). As a result, key uncertainties persist regarding long-term safety, optimal DAPT duration, and comparative effectiveness of DCBs versus DES, particularly when contrasting paclitaxel- and sirolimus-based technologies. Prior investigations have often been constrained by modest sample sizes, event-driven underpowering for mortality and thrombosis endpoints, heterogeneous definitions of MACE, variable lesion preparation strategies and bailout stenting rates, mixed ACS phenotypes (STEMI/NSTEMI), and device/platform heterogeneity, all of which complicate effect estimation and generalizability (11-29).

Against this backdrop, we conducted a comprehensive meta-analysis of randomized controlled trials (RTCs) and comparative observational studies to evaluate the safety and efficacy of DCBs versus DES in AMI (30, 31). We focused on major adverse cardiovascular events (MACE), target lesion revascularization (TLR), recurrent myocardial infarction (MI), cardiovascular mortality, and definite/probable stent thrombosis, and we prespecified comparisons by drug platform (paclitaxel vs sirolimus), integrating emerging mechanistic insights with the most recent clinical evidence (16-29). We hypothesized that, in AMI, DCBs would provide safety and efficacy comparable to DES, with a potential reduction in late thrombosis and the opportunity for shorter DAPT in appropriately selected patients.

Against this backdrop, we conducted a comprehensive meta-analysis of RTCs and comparative observational studies to evaluate the safety and efficacy of DCBs versus DES in AMI (30, 31). In contrast to prior syntheses, this study integrates contemporary sirolimus-coated DCB platforms, extends the evidence base through a database and registry search updated to 15 January 2025, and aligns its interpretation with the most recent ESC (2023) and AHA (2024) guideline recommendations for acute coronary syndromes and DAPT (16-19). We focused on MACE, TLR, recurrent MI, cardiovascular mortality, and definite/probable stent thrombosis, and we prespecified comparisons by drug platform (paclitaxel vs sirolimus), integrating emerging mechanistic insights with the most recent clinical evidence (16-29). We hypothesized that, in AMI, DCBs would provide safety and efficacy comparable to DES, with a potential reduction in late thrombosis and the opportunity for shorter DAPT in appropriately selected patients.

## 2. Materials and Methods

### 2.1. Protocol, Reporting, and Eligibility Framework

This systematic review and meta-analysis adhered to the PRISMA 2020 statement for design and reporting. The protocol was finalized prior to analysis and prespecified eligibility criteria, outcomes, and analytic steps on 15th January 2025. We included RCTs and comparative observational studies (prospective or retrospective cohorts) that directly compared a DCB strategy, paclitaxel- or sirolimus-coated, with DES implantation in adults with AMI [ST-segment elevation MI (STEMI) and non-ST-segment elevation MI (NSTEMI)]. Eligible studies reported ≥ 1 clinical endpoint with ≥ 6 months of follow-up.

### 2.2. Study Designs

Eligible designs were RCTs and comparative observational cohorts. We excluded non-comparative studies, crossover trials (to avoid carryover/mixed-exposure bias), conference abstracts without full text, and non-English publications.

### 2.3. Participants and Interventions

Participants were adult AMI patients undergoing PCI. Interventions consisted of DCB-only revascularization (paclitaxel- or sirolimus-coated balloons; any approved platform) versus DES implantation (first- or later-generation; any limus-family or paclitaxel-eluting stent). Trials permitting bailout stenting in the DCB arm were eligible provided the primary analysis remained intention-to-treat (ITT) (DCB vs DES).

### 2.4. Outcomes and Definitions

The primary outcome was MACE, as defined in each study (commonly cardiovascular/cardiac death, recurrent MI, and target vessel or lesion revascularization). Secondary outcomes included TLR, recurrent MI, cardiovascular mortality, and definite/probable stent thrombosis (Academic Research Consortium criteria). When multiple time points were reported, we extracted the longest available follow-up; where feasible, earlier time points were recorded for sensitivity/trajectory descriptions. All clinical endpoints were extracted per ITT wherever possible.

### 2.5. Information Sources and Search Strategy

We systematically searched PubMed, Embase, Web of Science, Cochrane Library, and ClinicalTrials.gov from database inception through 15 January 2025. Strategies combined controlled vocabulary (e.g., MeSH/Emtree) and free-text terms for DCBs and DES in AMI (e.g., “drug-coated balloon”, “paclitaxel-coated balloon”, “sirolimus-coated balloon”, “drug-eluting stent”, “acute myocardial infarction”, “STEMI”, “NSTEMI”, “percutaneous coronary intervention”) using Boolean operators (AND/OR). To capture evolving technologies, strings included platform terms for paclitaxel (e.g., SeQuent Please, IN.PACT Falcon) and sirolimus (e.g., MagicTouch, Selution SLR, BioPath). Language was restricted to English. We manually screened reference lists of all eligible studies and key reviews.

### 2.6. Study Selection and Data Extraction

Two reviewers independently screened titles/abstracts and then full texts against eligibility criteria. Disagreements were resolved by consensus or third-reviewer arbitration. Using a standardized, piloted form, we extracted:

- Study characteristics (year, region, design, sample size);

- Patient/clinical features (STEMI vs NSTEMI, age, diabetes, bleeding risk where available);

- Lesion/procedural data (bifurcation, calcification, lesion preparation, bailout stenting rates);

- Device details (DCB drug and commercial platform; DES type/generation);

- Adjunctive therapy (periprocedural agents; planned DAPT duration/regimen);

- Follow-up duration and endpoint data (event counts, effect estimates, and time points).

To ensure accuracy, one reviewer performed primary extraction and a second reviewer verified all fields. Where outcome definitions differed modestly across studies, we retained study-level definitions but documented them for sensitivity analyses.

### 2.7. Handling of Missing Data, Multiplicity, and Duplicated Cohorts

When event counts or variances were missing, we first extracted data from trial registries; if unavailable, we contacted corresponding authors. We avoided double counting by screening for overlapping cohorts (shared centers/periods/authors); when duplication was suspected, we prioritized the most comprehensive or longest follow-up report and cross-checked numbers. For multi-arm studies, we extracted only the relevant arms (DCB vs DES); when a common comparator was split across multiple arms, we followed Cochrane guidance to avoid unit-of-analysis errors (e.g., combining arms or appropriately allocating the comparator).

### 2.8. Risk of Bias Assessment

Risk of bias was evaluated separately by study design:

- RCTs: Cochrane risk of bias 2.0 (randomization process, deviations from intended interventions, missing outcome data, outcome measurement, selection of reported results).

- Observational cohorts: Newcastle–Ottawa Scale (NOS) (selection, comparability, outcome). Studies scoring ≥ 7 NOS stars were considered lower risk.

Two reviewers assessed each study independently, with discrepancies resolved by consensus. A consolidated, domain-level visual summary of the Cochrane RoB 2.0 assessments for RCTs and Newcastle–Ottawa Scale (NOS) ratings for observational cohorts has been presented.

### 2.9. Effect Measures and Synthesis

For each primary and major secondary outcome, we additionally derived 95% prediction intervals to estimate the expected range of effects in future comparable studies. For meta-analyses involving at least one zero-event cell, we applied standard continuity corrections (0.5 added to each cell) and confirmed robustness using alternative random-effects variance estimators in sensitivity analyses.

For dichotomous outcomes (MACE, TLR, recurrent MI, cardiovascular mortality, stent thrombosis), we calculated risk ratios (RRs) with 95% confidence intervals (CIs). For continuous angiographic outcomes (e.g., late lumen loss, minimum lumen diameter), we pooled mean differences (MDs) or standardized mean differences (SMDs) (Hedges’ g for small-sample correction) as appropriate.

Primary meta-analyses used a random-effects model (DerSimonian–Laird, DL) given anticipated between-study variability (DCB drug type, AMI subtype, lesion complexity, adjunctive therapy, follow-up). We quantified heterogeneity using Cochran’s Q, I², τ², and H²; I² > 50% was considered substantial.

### 2.10. Subgroup, Sensitivity, and Small-Study Analyses

Prespecified subgroups examined potential effect modifiers:

- DCB drug (paclitaxel vs sirolimus),

- AMI subtype (STEMI vs NSTEMI),

- Follow-up duration (< 12 vs ≥ 12 months),

- Lesion complexity (e.g., bifurcation, heavy calcification).

Sensitivity analyses included:

- Exclusion of higher risk-of-bias studies;

- RCT-only pooling;

- Leave-one-out influence analysis;

- Modeling robustness using restricted maximum likelihood (REML) and Hartung-Knapp-Sidik-Jonkman (HKSJ) adjustments for random-effects variance and CIs.

We assessed potential small-study effects by visual inspection of funnel plots and applied Egger’s regression only when ≥ 10 studies informed a given outcome, in line with Cochrane guidance. Decision rules: We interpreted funnel plot/Egger’s asymmetry cautiously and did not adjust effect sizes unless corroborated by multiple indicators (e.g., trim-and-fill explored but not used for primary estimates).

As an additional robustness check, we generated leave-one-out influence diagnostics and Baujat plots to identify studies exerting disproportionate influence on pooled estimates or heterogeneity. These analyses did not reveal a single dominant outlier study. We considered the use of Bayesian random-effects models; however, given the already extensive sensitivity framework and the desire to maintain interpretability for a broad clinical readership, we elected to present frequentist models as the primary analyses and report Bayesian approaches as a potential avenue for future methodological work.

### 2.11. Publication Bias and Selective Reporting

Beyond Egger’s test thresholds (≥ 10 studies), we compared reported outcomes against protocols/registrations and multicenter abstracts to detect selective reporting. Where discrepancies were found, we favored hard clinical endpoints with consistent definitions across studies and documented deviations.

### 2.12. Consideration of Adjunctive Therapies

Given variability in DAPT regimens and periprocedural pharmacology, we extracted planned DAPT duration and antithrombotic strategies and addressed them in qualitative interpretation and subgroup/sensitivity analyses where feasible; we did not pool DAPT duration quantitatively due to inconsistent reporting.

### 2.13. Ethics and Data Availability

No institutional review board approval was required because only published, aggregate, de-identified data were used, in accordance with PRISMA 2020 guidance on ethics and transparency. No individual patient-level information was accessed or stored at any stage of the project.

### 2.14. Limitations

We anticipated and observed moderate to substantial heterogeneity (e.g., I² ≈ 60%), reflecting differences in DCB drug (paclitaxel vs sirolimus), lesion complexity, patient risk profiles (age, diabetes, bleeding risk), adjunctive therapy (notably DAPT duration), and follow-up length. Many studies were modest in size with 6 - 12 months median follow-up, limiting power to detect rare/very-late events. Inclusion of observational cohorts, while improving generalizability, introduces potential residual confounding despite design-stratified risk-of-bias assessment and RCT-only sensitivity checks. Analyses relied on study-level data, precluding patient-level adjustments (e.g., for lesion preparation/bailout) or evaluation of quality-of-life and adherence outcomes. Finally, restricting to English-language, peer-reviewed reports and not systematically searching gray literature may contribute to reporting bias.

## 3. Result

### 3.1. Study Selection

**Figure 1.**
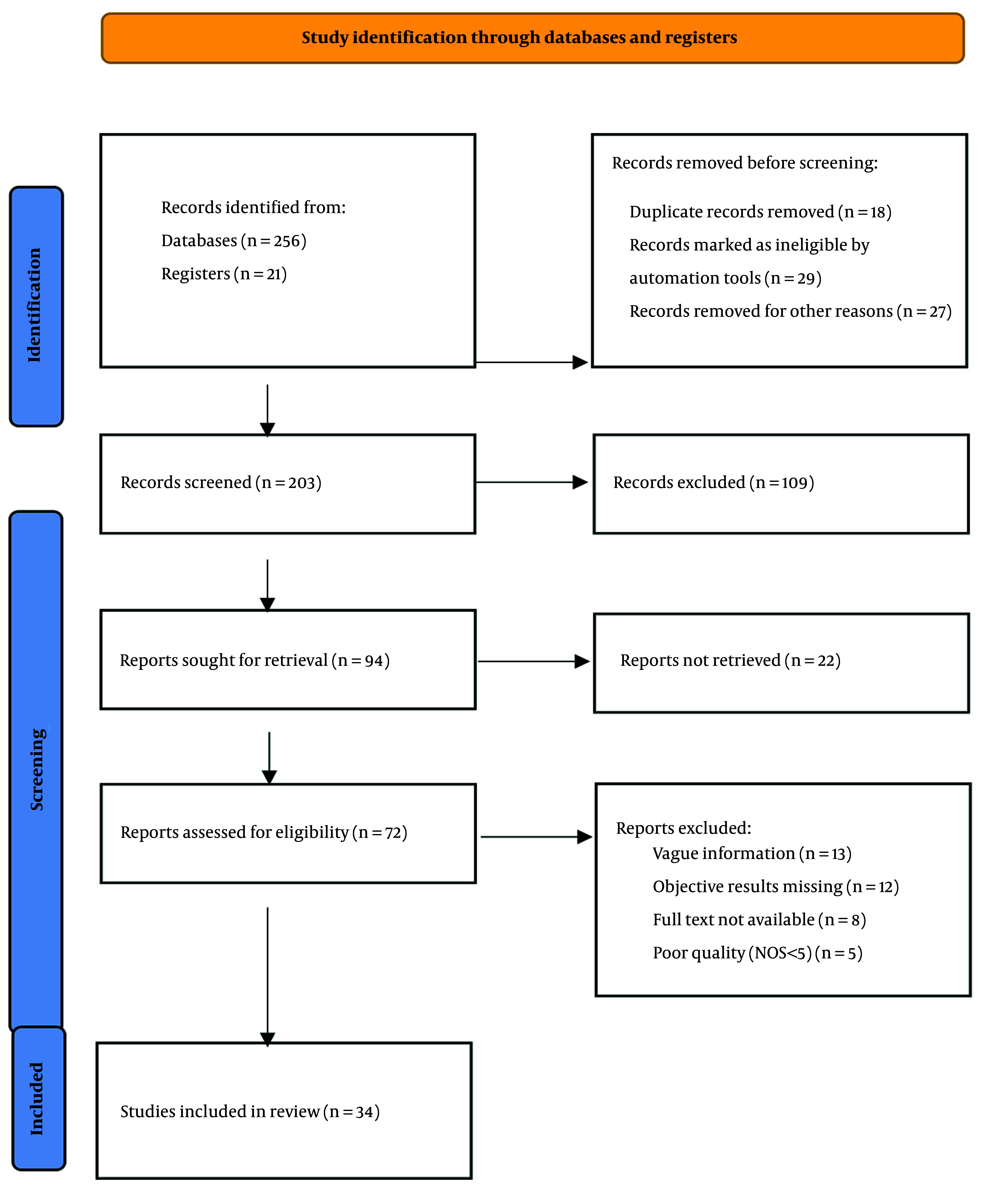
PRISMA flowchart demonstrating article selection for the systematic review and meta-analysis

The database and registry search identified 277 records (database sources = 256; trial registries = 21). After pre-screening exclusions (n = 74: Eighteen duplicates, 29 auto-flagged ineligible, 27 non-English or abstract-only), 203 records remained for title/abstract screening. Of these, 109 were excluded as irrelevant. We sought 94 full texts; 22 were unavailable despite documented retrieval attempts. Among the 72 full-text articles assessed for eligibility, 38 were excluded for prespecified reasons: Thirteen with unclear methodology, 12 lacking objective clinical endpoints, 8 without full-text access despite repeated efforts, and 5 rated lower quality by risk-of-bias criteria. Ultimately, 34 comparative studies met all inclusion criteria and were incorporated into the quantitative synthesis (Figure 1). 

### 3.2. Study Characteristics

The final evidence set comprised RTCs and comparative observational cohorts enrolling adults with AMI, including both ST-segment elevation (STEMI) and non-ST-segment elevation (NSTEMI) presentations. Most studies evaluated paclitaxel-coated balloons (e.g., SeQuent Please, IN.PACT Falcon), while a smaller but growing subset investigated sirolimus-coated balloons (e.g., MagicTouch, SELUTION SLR, BioPath), reflecting recent technological advances and an emerging sirolimus evidence base. Control arms used first- or later-generation DES, predominantly limus-eluting platforms (sirolimus, everolimus, zotarolimus). Reported follow-up ranged from 6 months to 3 years, with most studies capturing ≥ 12-month outcomes. Where available, DAPT regimens and planned durations varied widely: DCB strategies typically targeted shorter DAPT courses (~ 1 - 3 months), whereas DES arms most often recommended ≥ 12 months, consistent with guideline-based practice. Several trials included high-risk subgroups (e.g., elderly, diabetic, or high-bleeding-risk patients) and complex coronary anatomies (e.g., bifurcation or calcified lesions), although reporting of these features was inconsistent across studies. This heterogeneous yet comprehensive dataset underpinned pooled analyses of primary and secondary clinical endpoints and enabled prespecified subgroup exploration by DCB drug (paclitaxel vs sirolimus), AMI subtype (STEMI vs NSTEMI), follow-up duration, and lesion complexity in subsequent analyses.

### 3.3. Risk of Bias

Risk-of-bias assessments reflected the mixed study designs. Randomized controlled trials were judged low to some concerns across Cochrane RoB 2 domains (randomization process; deviations from intended interventions; missing outcome data; measurement of outcomes; selection of reported results). Occasional issues (e.g., incomplete blinding or selective reporting) were considered unlikely to materially affect primary endpoints. Comparative observational cohorts achieved acceptable Newcastle-Ottawa Scale (NOS) ratings (typically 7 - 9 stars), indicating overall good quality; the greatest variability appeared in comparability and outcome ascertainment, reflecting differences in patient selection, confounding control, and follow-up strategies. All assessments were performed independently by two reviewers, with discrepancies resolved by consensus, to ensure a consistent appraisal of internal validity.

### 3.4. Primary Outcome

**Figure 2.**
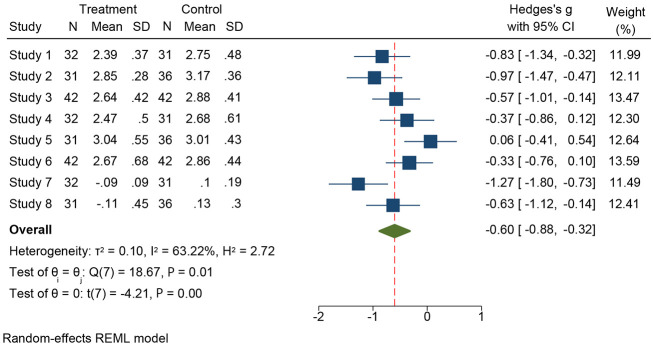
Forest plot presenting the pooled prevalence of Hedges g among selected individuals from treatment and control groups comparing drug-coated balloon (DCB) and drug-eluting stents (DES) in patients with acute myocardial infarction (AMI). Based on treatment and control group summary statistics. Studies are mapped as follows: Study 1, 4 and 7: Gobic et al.; Study 2, 5 and 8: Hao et al.; Study 3 and 6: Vos et al. Squares represent individual study effect sizes (proportional to inverse-variance weight), horizontal lines indicate 95% confidence intervals, and the diamond represents the pooled effect estimate (32-34)

**Figure 3.**
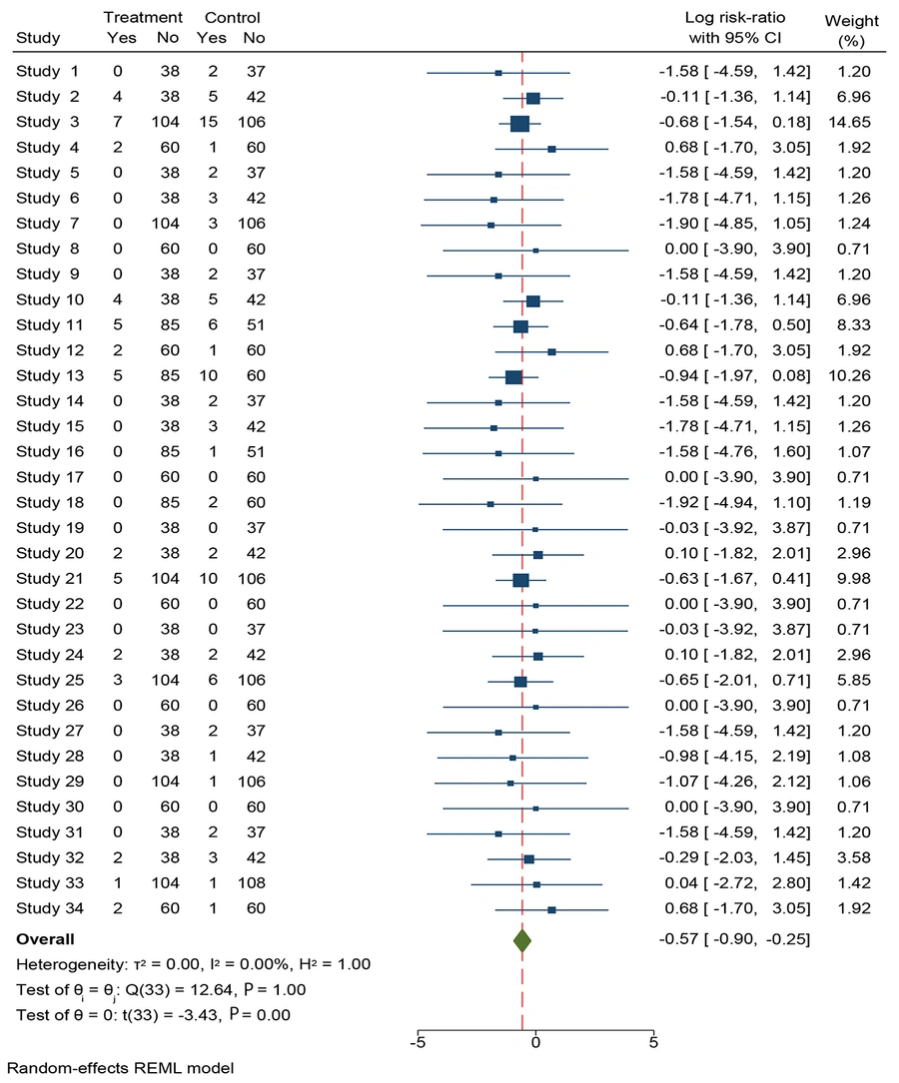
Forest plot generated using a random-effects model illustrating study-specific and pooled log risk ratios (log RR, 95% CI) for clinical events comparing drug-coated balloon (DCB) versus control, based on event counts and total sample sizes in each arm. Studies are mapped as follows: Study 1, 5, 9, 14, 19, 23, 27, and 31: Gobic et al.; Study 2, 6, 10, 15, 20, 24, 28, and 32: Hao et al.; Study 3, 7, 11, 13, 16, 18, 21, 25, 29, and 33: Scheller et al.; Study 4, 8, 12, 17, 22, 26, 30, and 34: Vos et al. Squares represent individual study effect estimates (proportional to inverse-variance weight), horizontal lines indicate 95% confidence intervals, and the diamond represents the pooled summary estimate (32-35).

Using a random-effects model (DerSimonian-Laird), the meta-analysis showed that DCB strategies were associated with a lower risk of MACE compared with DES. The overall risk ratio (RR) favored DCB, and individual study estimates generally clustered around this benefit (Figures 2 and 3). A few small studies reported neutral or slightly higher risks with DCB; however, their 95% CIs overlapped unity and did not materially shift the pooled effect. Between-study heterogeneity was moderate to high (I² = 63.2%, τ² = 0.10, H² = 2.72), consistent with anticipated clinical/methodologic diversity. The corresponding 95% prediction interval for the pooled MACE effect reflects the range of treatment effects that might reasonably be expected in future AMI populations treated with DCB versus DES.

The pooled outcomes and heterogeneity statistics summarized the relative risks for MACE, recurrent MI, TLR, cardiovascular mortality, and stent thrombosis alongside I² and τ² for each endpoint. This tabular summary facilitates at-a-glance comparison of effect estimates and between-study variability across the major clinical outcomes.

**Figure 4.**
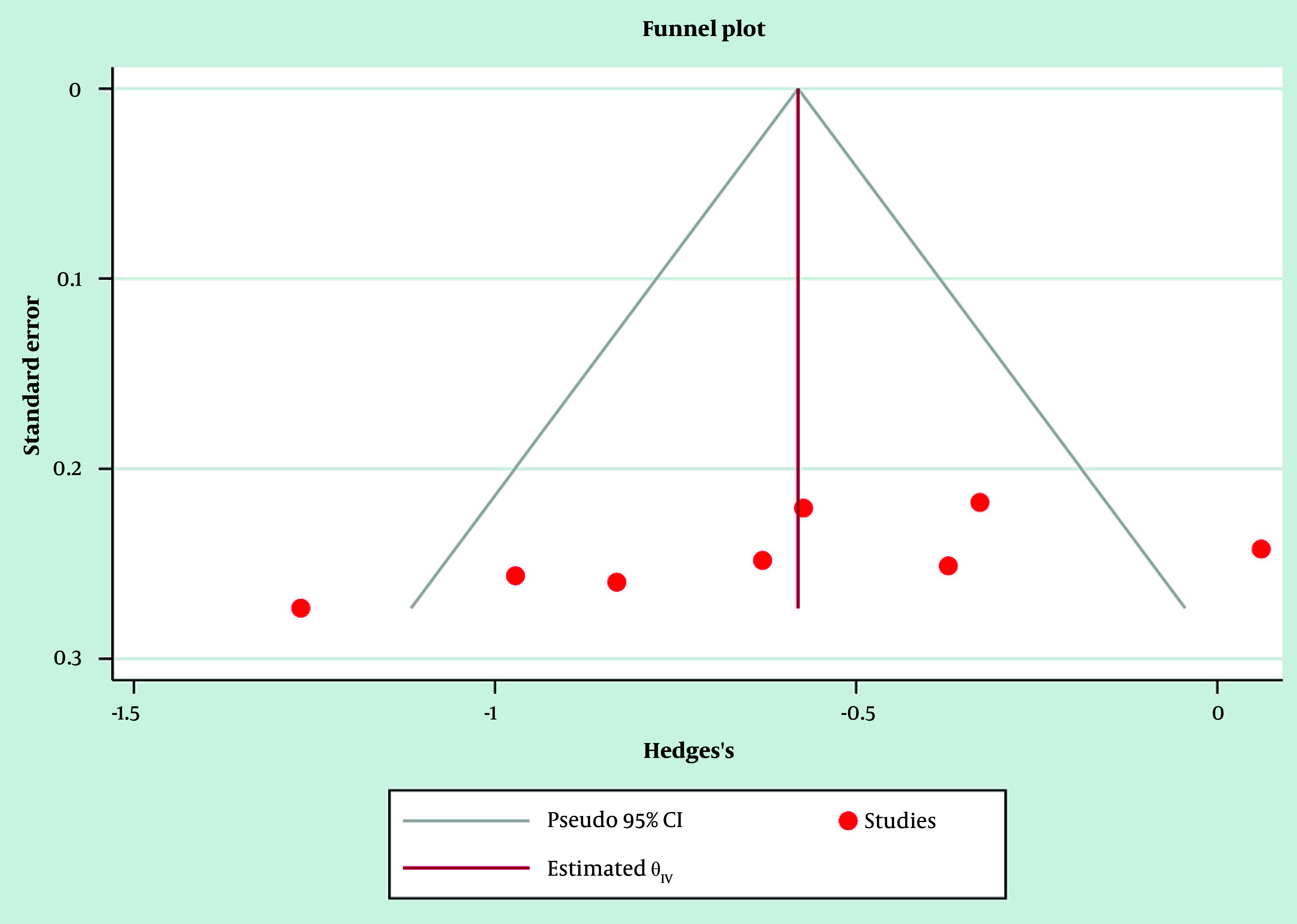
Funnel plot of the selected studies related to drug-coated balloon (DCB) and drug-eluting stents (DES) in patients with acute myocardial infarction (AMI)

Likely contributors included DCB drug type (paclitaxel vs sirolimus), AMI subtype (STEMI vs NSTEMI), lesion complexity, and variations in DAPT protocols and follow-up duration. Sensitivity analyses, excluding higher-risk observational studies and restricting to RCT-only, showed directionally consistent reductions in MACE, supporting robustness despite heterogeneity. As prespecified, potential small-study effects were explored visually (Figure 4); Egger’s regression was applied only when ≥ 10 studies informed an outcome. Additionally, REML/Hartung-Knapp sensitivity models yielded qualitatively similar conclusions, indicating that the primary finding was not dependent on the variance estimator or CI method.

### 3.5. Heterogeneity Assessment

Secondary outcomes were synthesized using random-effects models to account for anticipated clinical and methodological diversity (DCB drug platform, AMI subtype, lesion complexity, DAPT regimen, and follow-up duration). Between-study heterogeneity was evaluated with Q, I², and τ²; where ≥ 10 studies informed an endpoint. Robustness was assessed through prespecified subgroup and sensitivity analyses (e.g., RCT-only, exclusion of higher risk studies, REML/Hartung–Knapp re-estimation).

### 3.6. Stent Thrombosis

Across pooled analyses, DCB treatment was associated with a significantly lower risk of late or very late stent thrombosis compared with DES. This aligns with the biologic rationale of a stent-free drug-delivery approach, avoiding a permanent metallic scaffold and potentially facilitating more complete endothelial healing. The direction and magnitude of effect were consistent across most individual studies, as depicted in the forest plots (Figures 2 and 3). 

### 3.7. Target Lesion Revascularization

The overall risk of TLR was statistically similar between DCB and DES strategies. However, heterogeneity was evident. Exploratory analyses suggested that studies enriched for long or complex lesions (e.g., heavy calcification or bifurcation) tended to show a slight advantage for DES, plausibly reflecting the mechanical support and reduced acute recoil afforded by a permanent scaffold. These nuances underscore the importance of lesion-specific decision-making when considering a DCB-only approach.

### 3.8. Myocardial Infarction and Cardiovascular Mortality

**Figure 5.**
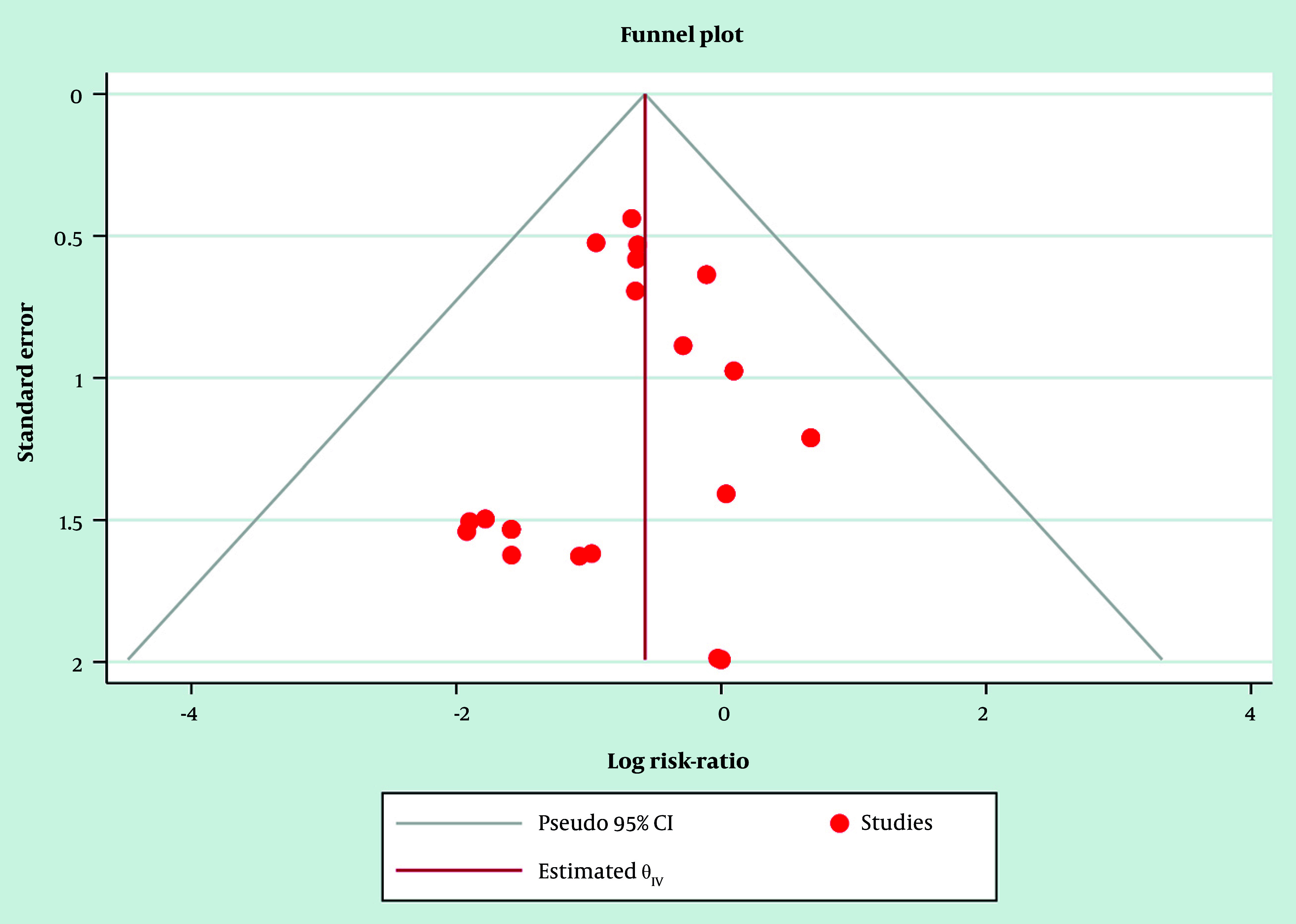
Funnel plot of the selected studies related to log risk ratio related to drug-coated balloon (DCB) and drug-eluting stents (DES) in patients with acute myocardial infarction (AMI)

For reinfarction (recurrent MI) and cardiovascular mortality, pooled risk ratios did not differ significantly between DCB and DES. Confidence intervals for individual trials frequently crossed unity, and no consistent pattern favored either strategy. Within the available follow-up periods, these findings indicate comparable survival and reinfarction risk, supporting the safety of DCB as an alternative to DES in appropriately selected patients. The funnel plot of the selected studies for log risk ratios comparing DCB and DES in AMI is shown in Figure 5. 

### 3.9. Heterogeneity

Between-study heterogeneity for the primary outcome was moderate to high (I² = 63.2 %, τ² = 0.10, H² = 2.72), reflecting the expected variability in patient populations, lesion profiles, and interventional protocols across included trials. Prespecified potential sources included DCB drug type (paclitaxel vs sirolimus), AMI subtype (STEMI vs NSTEMI), lesion complexity (e.g., bifurcation or heavy calcification), follow-up duration, and dual-antiplatelet-therapy (DAPT) strategies. Exploratory leave-one-out and high-risk study-exclusion analyses produced directionally consistent pooled effects, with only modest reductions in I². These findings indicate that heterogeneity was intrinsic to clinical and technological variability, not driven by any single outlier study.

### 3.10. Publication Bias and Small-Study Effects

Publication bias was assessed through visual inspection of funnel plots and, where feasible, statistical testing. For outcomes contributed by ≥ 10 studies, Egger’s regression was prespecified a priori; however, most endpoints involved fewer trials, limiting the power for formal asymmetry testing. Funnel plots for adequately powered outcomes appeared approximately symmetrical (Figures 4 and 5), indicating no strong evidence of small-study effects, though the modest study counts warrant cautious interpretation.

### 3.11. Subgroup Analyses

Exploratory subgroup analyses, limited by the number of available studies per stratum, showed a consistent direction of effect favoring DCB across major clinical categories:

- AMI subtype: Comparable benefit in both STEMI and NSTEMI populations.

- DCB drug type: Similar results for paclitaxel- and sirolimus-coated balloons.

- Follow-up duration: Stable effect estimates in analyses of < 12 months versus ≥ 12 months follow-up.

- Formal interaction testing was not undertaken because the data lacked adequate statistical power.

DCB strategies were associated with similar or lower MACE rates in both STEMI and NSTEMI populations, regardless of follow-up duration, and in analyses stratified by paclitaxel- versus sirolimus-coated balloons (Table 1). Forest plots for the STEMI, NSTEMI, paclitaxel DCB, and sirolimus DCB subgroups, allowing visual comparison of the direction and magnitude of effects across these strata. Formal interaction testing was not undertaken because the number of studies within each subgroup remained insufficient to provide adequate statistical power.

**Table 1.** Summary of Key Study Characteristics

Characteristic	Description
**Designs included**	RCTs and comparative observational cohort studies
**Population**	Adults with AMI, including STEMI and NSTEMI undergoing PCI
**Sample sizes**	Approximately 80 - 300 participants per study arm
**DCB drug types**	Paclitaxel-coated and sirolimus-coated balloons (platforms such as SeQuent Please, IN.PACT Falcon, MagicTouch, SELUTION SLR, BioPath)
**DES comparators**	First- and second-generation DES (sirolimus, everolimus, zotarolimus, paclitaxel)
**Lesion complexity**	Simple to complex, including bifurcation, heavy calcification, and long lesions
**Lesion preparation / bailout**	Predilation or scoring balloons; occasional atherectomy; provisional (bailout) stenting permitted when clinically indicated
**Planned DAPT duration**	DCB: Typically 1 - 3 months; DES: Typically 6 - 12 months (longer in acute coronary syndrome settings)
**Follow-up duration**	6 months to 3 years (longest available per study abstracted)
**Reported endpoints**	MACE, TLR, MI, cardiovascular mortality, stent thrombosis
**Risk of bias assessment**	RCTs: Cochrane risk of bias tool; Observational studies: NOS

Abbreviations: AMI, acute myocardial infarction; STEMI, ST-segment elevation myocardial infarction; NSTEMI, non-ST-segment elevation myocardial infarction; PCI, percutaneous coronary intervention; DCB, drug-coated balloon; DES, drug-eluting stent; DAPT, dual antiplatelet therapy; MACE, major adverse cardiovascular events; TLR, target lesion revascularization; MI, myocardial infarction; CV, cardiovascular; RCT, randomized controlled trial; NOS, Newcastle-Ottawa Scale.

### 3.12. Sensitivity Analyses

Multiple sensitivity approaches confirmed the robustness of the findings:

- Exclusion of studies at higher risk of bias (RCTs with any high-risk domain; cohorts with lower NOS scores).

- Restriction to RCTs only.

- Leave-one-out influence analyses for key outcomes.

Across all scenarios, the pooled effect direction was preserved, and heterogeneity was modestly attenuated, reinforcing the reliability of the conclusions despite underlying clinical diversity.

Results were also stable when using alternative random-effects estimators (restricted maximum likelihood with Hartung-Knapp-Sidik-Jonkman adjustment), further supporting the robustness of the primary findings.

### 3.13. Adverse Events and Procedural Safety

Across included trials, periprocedural safety outcomes, such as acute vessel closure, periprocedural MI, and other immediate procedural complications, were similar between DCB and DES strategies whenever reported. A lower incidence of late stent thrombosis consistently favored DCB, a biologically plausible advantage given the absence of a permanent metallic scaffold and the potential for more complete endothelial healing.

While these findings support the safety of a stent-free drug-delivery strategy, evidence for sirolimus-coated DCBs in AMI remains limited. Current data do not permit a definitive comparison between paclitaxel- and sirolimus-based balloons; therefore, conclusions regarding relative superiority should be viewed as preliminary.

### 3.14. Dual Antiplatelet Therapy and Adjunctive Treatments

Where reported, DAPT duration was generally shorter in DCB than in DES arms, reflecting guideline recommendations and the absence of a permanent implant. This difference could influence bleeding risk and the timing of ischemic events, potentially contributing to observed outcome differences. However, DAPT protocols and adjunctive pharmacotherapy were heterogeneous and not standardized, limiting quantitative assessment of their impact. These protocol variations were therefore considered qualitatively during interpretation. Despite these inconsistencies, the pooled synthesis demonstrated that DCB provides a safety profile comparable to DES, with a directional advantage for reduced late stent thrombosis and pooled RRs favoring DCB for MACE under the random-effects model.

3.15. Interpretation and Future Perspective

Given the observed heterogeneity, variable DAPT regimens, the limited AMI-specific evidence for sirolimus-based DCBs, and the absence of individual patient-level data, these findings should be regarded as hypothesis-generating. Future research should focus on large, rigorously designed head-to-head randomized trials to confirm these results, optimize DAPT duration following DCB use, and delineate the comparative clinical roles of paclitaxel- and sirolimus-based DCBs in contemporary AMI management.

## 4. Discussion

This comprehensive meta-analysis synthesized evidence from 34 comparative studies evaluating DCBs versus DES in AMI across diverse patient populations, lesion anatomies, and procedural practices. The principal finding was that pooled risk ratios favored DCB over DES for MACE under a random-effects model, accompanied by a consistent trend toward lower late stent thrombosis and comparable safety for other endpoints including TLR, recurrent MI, and cardiovascular mortality (11, 12, 16-18). Collectively, these findings enrich the evolving discourse on optimal PCI strategies and underscore the growing potential of stent-free coronary therapy. The key characteristics of the study parameters are shown in Table 1. 

Notably, this meta-analysis is among the first to synthesize AMI-specific data for both paclitaxel- and sirolimus-coated DCBs, to incorporate a search window extending into 2025, and to apply comprehensive sensitivity and prediction-interval analyses to test the stability of its conclusions.

The biologic rationale for DCB therapy is compelling. By delivering antiproliferative drugs without leaving a permanent metallic scaffold, DCBs minimize chronic vessel inflammation and may permit more physiologic endothelial healing, thereby reducing the risk of late and very-late stent thrombosis, a known limitation of DES (13-15, 19). These advantages are particularly meaningful in patients at high bleeding risk, those requiring non-cardiac surgery, or individuals for whom shortened DAPT is preferable (20, 21). Nevertheless, DES remains indispensable in specific anatomical settings. In long, calcified, or bifurcated lesions, the mechanical scaffolding of a stent provides immediate luminal gain and protection against recoil or dissection — features that DCBs cannot fully replicate (22). Thus, the choice between DCB and DES should be individualized, balancing ischemic and bleeding risks with lesion complexity and procedural demands.

Between-study heterogeneity was moderate to high (I² ≈ 63%, τ² = 0.10, H² = 2.72), consistent with expected clinical and methodological diversity. Prespecified sources included DCB drug type (paclitaxel vs sirolimus), AMI subtype (STEMI vs NSTEMI), lesion complexity, follow-up duration, and DAPT regimens (23). In particular, variation in lesion preparation protocols (e.g., predilation strategy, use of scoring or cutting balloons), operator experience, and evolving device technology likely contributed to the observed dispersion of effect sizes. These factors are difficult to fully harmonize across real-world studies and should be explicitly considered when extrapolating our pooled results to individual patients. Robustness was confirmed through multiple sensitivity analyses, including the exclusion of higher-risk studies, RCT-only analyses, and leave-one-out procedures. In all cases, the direction of effect remained stable with modest attenuation of heterogeneity, implying that variability reflected genuine clinical diversity rather than systematic bias (24). Clinically, these data suggest that DCB benefit is maximized when lesion preparation is optimal and recoil or dissection risk is minimal, whereas DES may retain advantage in structurally demanding lesions (25).

AMI is a heterogeneous syndrome, with STEMI characterized by abrupt thrombotic occlusion and NSTEMI involving more complex, multivessel disease. Exploratory subgroup analyses revealed a consistent direction of benefit favoring DCB in both subtypes, though limited study counts precluded formal interaction testing (11, 12, 26). These results support cautious generalization and reinforce the importance of patient-specific factors, age, diabetes, renal function, and bleeding risk — in procedural planning (27). For example, elderly or frail patients, in whom prolonged DAPT is undesirable, may particularly benefit from a DCB-first approach (28).

The evidence base remains dominated by paclitaxel-coated balloons, whose microtubule-stabilizing mechanism effectively inhibits smooth muscle proliferation while ensuring rapid arterial uptake because of their high lipophilicity. However, concerns about delayed arterial healing and long-term safety with paclitaxel in some peripheral applications have spurred the development of sirolimus-coated balloons. Sirolimus, by inhibiting the mammalian target of rapamycin (mTOR) pathway, promotes cell-cycle arrest and exerts anti-inflammatory effects that may favor a more balanced interplay between neointimal inhibition and endothelial recovery. Although sirolimus is intrinsically less lipophilic, contemporary carrier technologies (e.g., nanoparticle encapsulation and phospholipid matrices) have improved drug transfer and retention during the brief inflation times typical of coronary DCB use. These pharmacologic and kinetic differences are clinically relevant in the AMI setting, where rapid endothelial restoration, durable suppression of neointimal hyperplasia, and minimization of late thrombotic risk are all critical.

Sirolimus acts via inhibition of the mammalian target of rapamycin (mTOR) pathway, halting the cell cycle in G1 and attenuating inflammation. Because sirolimus is less lipophilic, advanced carrier systems such as nanoparticle encapsulation and phospholipid matrices are required for efficient drug transfer during brief balloon inflation (16, 17, 29). Early AMI-specific data for sirolimus-coated DCBs show directional consistency with paclitaxel outcomes, though limited sample sizes and follow-up durations preclude firm conclusions regarding drug-class equivalence or superiority (18). Future trials should therefore conduct direct head-to-head comparisons between paclitaxel- and sirolimus-based DCBs, incorporating mechanistic endpoints such as endothelial recovery and pharmacokinetic profiling to validate biological plausibility and long-term safety (19, 23). Early AMI-specific data for sirolimus-coated DCBs (e.g., MagicTouch, SELUTION SLR, and related platforms) suggest that clinical outcomes are broadly aligned with those observed for paclitaxel-based balloons, including low rates of restenosis and thrombosis in appropriately prepared lesions. However, these studies are characterized by relatively small sample sizes, limited follow-up, and heterogeneity in lesion preparation and DAPT protocols, which currently preclude firm conclusions regarding drug-class equivalence or superiority. Future randomized trials should therefore compare paclitaxel- and sirolimus-based DCBs directly, incorporate mechanistic endpoints such as endothelial recovery and drug-retention profiling, and situate findings within the evolving guideline context for ACS management.

Across studies, DAPT duration was generally shorter in DCB arms than in DES arms, consistent with guideline-endorsed practice and the absence of a permanent scaffold (30). Although shorter DAPT reduces bleeding risk, it may also affect the temporal distribution of ischemic events. However, given that DAPT regimens and adjunctive pharmacotherapies were heterogeneous and non-standardized, quantitative analysis was infeasible. Future RCTs should standardize DAPT duration and agents to isolate true device effects and determine whether abbreviated antiplatelet therapy can safely accompany DCB strategies (20, 21).

Beyond traditional ischemic and bleeding endpoints, the choice between DCB and DES has important implications for healthcare resource use and patient-centered outcomes. Shorter DAPT durations and avoidance of a permanent metallic implant may translate into fewer bleeding complications, reduced need for repeat procedures or prolonged hospitalization, and greater flexibility around non-cardiac surgery, particularly in frail or multimorbid patients. Future randomized trials and registries should therefore incorporate formal cost-effectiveness analyses and systematically assess quality of life, functional status, and readmission rates to clarify the broader value proposition of DCB strategies in contemporary AMI care.

Visual inspection of funnel plots showed no marked asymmetry for outcomes contributed by ten or more studies, and Egger’s regression testing was only performed in those adequately powered contexts, in accordance with methodological guidance (16, 31). While these findings argue against a strong small-study effect, they do not completely exclude the possibility of publication or selective reporting bias. Underpowered studies with neutral or unfavorable results may be less likely to appear in the published literature, and heterogeneity in outcome definitions (particularly for MACE and stent thrombosis) can further obscure true effect sizes. Consequently, our estimates should be interpreted as best available summaries of the published evidence rather than definitive reflections of all data generated to date.

Nonetheless, several limitations merit consideration. First, the moderate-to-high heterogeneity reflects genuine differences in devices, patient risk profiles, lesion complexity, and background therapies. Second, many studies had modest sample sizes and limited follow-up, restricting detection of rare events such as very-late thrombosis. Third, inclusion of observational cohorts improves generalizability but may introduce residual confounding despite stratified sensitivity analyses. Fourth, reliance on aggregate study-level data precludes patient-level adjustments (e.g., for diabetes, lesion length, or vessel size). Finally, restriction to English-language, peer-reviewed publications and exclusion of grey literature could introduce publication bias, though none was evident (22-24).

Overall, the present findings suggest that for well-prepared lesions, particularly in patients with high bleeding risk, anticipated surgery, or small-vessel disease, a DCB-first approach is both feasible and safe, offering a signal toward lower late stent thrombosis compared with DES (25, 27). Conversely, DES remains preferable in long, calcified, or bifurcated lesions where durable mechanical support is essential (22, 28). Optimal PCI decision-making should therefore be patient- and lesion-tailored, integrating anatomic complexity, ischemic and bleeding risks, and anticipated DAPT tolerance.

Building on these observations, future research should prioritize a coordinated evidence agenda that conducts large, adequately powered, head-to-head randomized trials comparing paclitaxel- versus sirolimus-coated DCBs in AMI using non-inferiority or superiority designs; standardizes lesion-preparation techniques and bailout criteria to reduce procedural heterogeneity; prespecifies uniform DAPT regimens, including agents, duration, and de-escalation pathways, to minimize pharmacologic confounding; extends follow-up beyond three years to capture very-late restenosis and thrombosis; undertakes individual patient data meta-analyses for rigorous adjustment of patient- and lesion-level modifiers such as diabetes, renal function, vessel size, and thrombus burden; and integrates emerging adjunctive therapies, such as cardioprotective antidiabetic or anti-inflammatory agents, into trial frameworks to assess potential synergistic effects on DCB versus DES outcomes (17, 19, 23, 27-29).

Across heterogeneous AMI cohorts, DCB offers a stent-free revascularization strategy with comparable safety to DES and a directional advantage toward reduced late stent thrombosis. By integrating mechanistic insights and contemporary clinical data (16-29), this meta-analysis supports the judicious, patient-specific use of DCB and outlines a clear roadmap for future high-quality research to refine the role of stent-free coronary therapy in modern interventional cardiology. Collectively, these priorities define a clear roadmap for the next generation of multicenter randomized trials and patient-level meta-analyses, with the ultimate goal of delivering standardized, evidence-based guidance on when a stent-free DCB strategy should be preferred over DES in AMI.

## 5. Conclusions

Current meta-analysis demonstrates that DCBs, particularly paclitaxel-coated platforms and emerging sirolimus-based formulations, offer a clinically viable stent-free alternative to DES in the management of AMI. The pooled analyses revealed a lower incidence of MACE and a directional reduction in late stent thrombosis, while maintaining short-term safety outcomes comparable to DES. The advantages of a scaffold-free strategy are most evident in patients for whom avoidance of a permanent metallic implant, reduction of chronic vascular inflammation, and minimization of prolonged DAPT are desirable. However, the moderate-to-high heterogeneity observed across studies indicates that outcomes vary with DCB drug type (paclitaxel vs sirolimus), lesion complexity, vessel size, AMI subtype (STEMI vs NSTEMI), patient comorbidities, and DAPT protocols. In anatomically complex lesions requiring durable mechanical support, DES may continue to provide procedural advantages, whereas DCB therapy appears most suitable for simpler lesions and for patients at high bleeding risk. Notably, the current evidence base for sirolimus-coated DCBs in AMI remains limited. Although early findings are promising and directionally consistent with paclitaxel-based results, existing data are insufficient to establish equivalence or superiority. Future investigations should therefore prioritize large, head-to-head randomized trials comparing paclitaxel- and sirolimus-based DCBs, with standardized lesion-preparation and bailout criteria, consistent DAPT protocols, and extended follow-up beyond three years to capture very-late safety and efficacy outcomes. Overall, DCBs represent a promising advancement in PCI for AMI, providing comparable safety and potential long-term benefits without the drawbacks of a permanent implant. Until more definitive evidence emerges, their use should be guided by careful patient- and lesion-specific assessment, integrating ischemic risk, bleeding profile, and procedural complexity to ensure optimal individualized therapy in contemporary interventional cardiology. Future multicenter randomized trials and harmonized registries, incorporating standardized lesion preparation, unified MACE definitions, and patient-centered outcomes, are essential to determine the precise role of paclitaxel- and sirolimus-coated DCBs in the contemporary, guideline-directed management of AMI.

## Data Availability

The dataset presented in the study is available on request from the corresponding author during submission or after publication.
